# A Poly-Aromatic Hydrocarbon-Grafted Silicon-Quantum-Dot-Based Desorption Agent with High Salt Resistance and Its Influence on the Adsorption/Desorption Behavior of CBM in Deep Coal Rock

**DOI:** 10.3390/polym18070803

**Published:** 2026-03-26

**Authors:** Haibo Li, Lei Yue, Hongxing Xu, Yanhong Li, Yining Zhou, Rong Zhang, Kongjie Wang, Hongzhong Zhang, Shuai Luo, Bin Ren, Fei Chen, Yufei Liu

**Affiliations:** Changqing Downhole Technology Company, CNPC Chuanqing Drilling Engineering Company Limited, Xi’an 710018, China; lihaib@cnpc.com.cn (H.L.); cj_liyanh@cnpc.com.cn (Y.L.); cj_zhouyn@cnpc.com.cn (Y.Z.); cqjx_zhrong@cnpc.com.cn (R.Z.); cj_wangkj@cnpc.com.cn (K.W.); zhanghongzh@cnpc.com.cn (H.Z.); cj_luoshuai@cnpc.com.cn (S.L.); renbin@cnpc.com.cn (B.R.); cqjx_chenf@cnpc.com.cn (F.C.); cj_liuyf@cnpc.com.cn (Y.L.)

**Keywords:** deep coal-bed gas, desorption agent, modified silicon quantum dot, isothermal adsorption, wettability alteration

## Abstract

Coal-bed gas well production is too low to realize a highly efficient exploitation of the #8 coal seam in the Shanxi formation in the Nalin region. Based on the reservoir characteristics, the designed poly-aromatic-grafted silicon-quantum-dot-based desorption agent (PQS) has been developed. Then, the adsorption/desorption behavior of CBM on the coal surface under the influence of this active chemical has been studied, and the synergy effect with an anionic–nonionic surfactant to desorption of CBM has also been discussed. The results show that the developed poly-aromatic-grafted silicon quantum dot, with a median size of 4.9 nm and +5.6 mV of zeta potential in neutral condition, has a significant emission peak with 470 nm at the excitation of 380 nm and 150,000 mg/L of salinity resistance, which also generates a strong adsorption capacity on the coal surface. A promoting effect to desorption of CBM for PQS nanofluid is exhibited and the Langmuir pressure is obviously increased. However, when the PQS nanofluid is synergized with an anionic–nonionic surfactant, the desorption of CBM is further improved and the wettability of the coal surface is altered from 78.2° to 84.2°. The desorption rate for this compound system reached 65.3%. It can be found that combining the quantum size, π–π stacking, π–π conjugation, and the synergy effect between PQS nanofluid and surfactant fluid with the traditional intermolecular force has a stronger capacity for promoting desorption of CBM than the conventional desorption agent. This study provides guidance for the molecular design of the desorption agent for deep coal rock and the application of silicon quantum dots.

## 1. Introduction

In recent years, research hotpots for unconventional oil and gas development have concentrated on deep coal-bed gas with over 2000 m of depth. In the #8 coal seam in the Shanxi formation in the Nalin region, with a depth of over 3000 m, methane mainly exists in two ways, including absorbing on the surface of the coal-bed via inter-molecular forces and free storage in pores and fractures [1,2,3]. When channels have been created between the coal-bed formation and the wellbore via hydraulic fracturing, the free gas and formation water flow out and into the wellbore. Then, the reservoir pressure has been decreased and part of the absorbed gas can be moved and desorb from the coal-bed surface until the reservoir pressure is lower than the desorption pressure. Even so, there will still be a mass of coal-bed gas in the reservoir, the further development of which requires alternative methods [4,5,6]. Some chemicals such as surfactants and polymers have a competitive absorption effect with methane on the surface of coal-bed. Therefore, these chemicals, with a stronger inter-molecular force on the coal rock surface, are injected into the reservoir to compel methane to desorb. 

It is a very hot technology for enhancing the recovery of coal-bed gas via this chemical method. Li et al. investigated wetting properties and methane adsorption/desorption characteristics and the effect of surfactants (STS). Their results showed that the coal rock treated with the wettability reversal agent had a larger methane adsorption capacity. The water-containing coal rock samples had a weaker adsorption capacity than the air-dried samples. In other words, the hydrophilicity was better and the methane adsorption was weaker [7]. Gurses et al.’s results showed that the adsorption of CTAB onto an active carbon/water interface mainly took place via ion exchange, ion pairing, and hydrophobic bonding [8]. You et al.’s results showed that compared with a conventional anionic surfactant and a cationic surfactant, the nonionic surfactant, N-(2-hydroxypropyl) perfluorooctane amide (FCS), exhibited lower Langmuir pressure during the entire CBM desorption process, which promoted the desorption of CBM. The nonionic surfactant FCS had the weakest adsorption capacity on the coal surface and increased the contact angle slightly, which enhanced hydrophobicity [9]. Ji et al.’s results showed that a higher chemical activity was demonstrated for [HOEmim][Cl] due to the presence of the –OH group. Compared to untreated coal, [HOEmim][Cl] exhibited a greater ability than [Emim][Cl] to improve the wetting properties of coal [10]. Chen X et al.’s results showed that compared with G526 and D001, after NH766 treatment, the decrease in the content of oxygen-containing functional groups in coal rock is the largest, by 30%, and the contact angle of the coal matrix surface increased by 10°. Furthermore, its hydrophobicity was enhanced, and the desorption amount increased by 24% [11]. Qi Y et al.’s results showed that hydrophobic CZH-1 surfactant leads to a significant increase in the desorption rate of CBM. The invasion of the hydrophobic CZH-1 surfactant resulted in decreases in the total area of the C–O peak of the oxygen-containing functional group and in the hydrophilic point in the coal [12]. Jia L et al.’s results showed that the imbibed water decreased by 59.99% and gas-flooding water recovery was enhanced by about 20.31% after the wettability of the coal sample was altered from water-wetness to gas-wetness [13].

It can be found that researchers usually design the desorption agent from the three key performance parameters, which include surface tension, reservoir wettability alteration, and competitive absorption. Capillary resistance can be obviously decreased by a desorption agent via reducing the surface tension and improving the surface wettability of the coal-bed to be more hydrophobic, which apparently increases the permeability of coal-bed gas. Then, the desorption agent with strongly competitive absorption increases the desorption pressure of coal-bed gas, which decreases the discharge threshold of the coal-bed gas. Unfortunately, the traditional desorption agent used for competitive absorption mainly relies on the oxygen-containing groups in the molecular structure and coal-bed surface via electrostatic effect, hydrogen bonding, and Van der Waals force. However, this designed conception is mainly for the competitive absorption on the sand rock surface. There is a significant difference between the sand rock and coal-bed in the mineral composition and the functional groups of the surface molecular structure. Therefore, in this paper, a poly-aromatic-grafted silicon quantum dot has been developed aiming to improve desorption of CBM in #8 coal seam in Shanxi formation in Nalin region. Then, adsorption behavior for it on the surface of coal powder and its influence to the adsorption/desorption behavior of CBM have been studied. Furthermore, the wettability alteration behavior is also discussed. 

Combining with the targeted reservoir characteristics, the design theory of PQS is mainly from the four aspects of the basic characteristics of coal rock, quantum size effect, intermolecular force, and synergistic effect, which is described as follows:

The component of the #8 coal seam mainly contains more than 85% of organics, mainly vitrinite, and bits of other inorganic components, mainly clay. Furthermore, the vitrinite has a large amount of aromatics. Therefore, there is a significant difference in the intermolecular force with the chemicals on the surface between coal rock and sand rock due to the significant variation of functional groups. Consequently, unlike the strong binding energy of the emphasized hydrogen bonds on the traditional desorption agent for sand rock, the emphasized poly-aromatic hydrocarbons are grafted on the surface of silicon quantum dots to increase the binding energy on the surface of coal rock via π–π stacking interaction and π–π conjugate effect [14,15].

The #8 coal seam in Nalin region has a high salinity of production water with at least 50,000 mg/L. To ensure the salinity resistance of desorption agent, the sulfonyl betaine functional group is added in the design of molecular structure, which ensures the strong anti-salt performance [16,17]. 

To enhance the binding energy of hydroxyl groups on the surface of clay in coal rock, the oxygen-containing functional groups are also adopted in the molecular structure, which builds hydrogen bond to improve the desorption effect [18,19]. 

In addition, because of the existence of negative charge on the surface of coal rock, amino cationic monomer is employed to strengthen the binding energy via the electrostatic attraction [20,21]. 

Moreover, quantum dot nanoparticles have super-low size and super-high specific surface energy to adsorb on the surface of other substances to realize the interface stability due to their size effect, which is a key reason for the application of nanoparticles in this desorption agent to increase its binding energy on the surface of coal rock [22,23].

Furthermore, it has been verified that an excessive osmotic pressure is formed by nanofluid in the wedge region between the bubble and the solid surface to compel desorption of gas. Therefore, the nanoparticle and surfactant fluid have an excellent synergy to powerfully separate the CBM from the surface of coal rock and higher desorption performance than the pure surfactant fluid or the pure nanofluid [24,25]. However, the #8 coal seam has a low permeability, less than 1 mD. For this case, the quantum dots with the significant advantage of super-low size will bring stronger osmotic pressure and can go into a narrower pore to realize the longer migration than the conventional nanoparticle [26,27,28], which has an excellent synergistic effect to form a strong competitive adsorption capacity to promote desorption of CBM.

**Figure 1:**
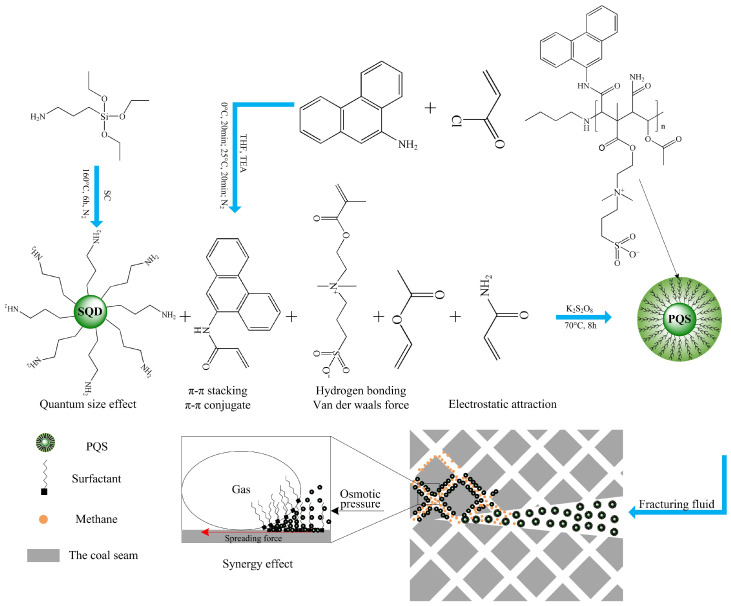
The schematic diagram for the design theory of poly-aromatics-grafted silicon quantum dot-based desorption agent and desorption mechanism.

As a result, the in situ polymerization is used to make the monomers with an aromatic ring, the sulfonyl betaine functional group, the oxygen-containing functional group, and amino cationic to be grafted on the surface of silicon quantum dots. It combines π–π stacking interaction, π–π conjugation, hydrogen bond, Van der Waals force, electrostatic attraction, and quantum size effect to form strong competitive adsorption with CBM on the surface of coal rock. Meanwhile, this modified silicon quantum dot nanofluid has excellent anti-salt activity because of its zwitterionic molecule structure and synergies with the oxygen-containing anionic–nonionic surfactant to forcefully enhance the desorption capacity of CBM. Figure 1 is the schematic diagram for the design theory of poly-aromatics-grafted silicon quantum dot-based desorption agent and desorption mechanism.

## 2. Materials

Sodium citrate (SC, 99.99%), (3-aminopropyl)triethoxysilane (APTES, 99.99%), Tetrahydrofuran (THF, 99.99%), 9-phenanthrylamine (99.99%), Trolamine (TEA, 99.99%), Acryloyl chloride (99.99%), N,N-dimethyl acetamide (99.99%), Sulfobetaine methacrylate (99.99%), Vinyl acetate (99.99%), Acrylic amide (99.99%), Potassium persulfate (99.99%) are all purchased from Aladdin (Shanghai, China). Glycerol (99%) and D-sorbitol (99%) are purchased from Macklin (Shanghai, China). Aliphatic alcohol polyoxyethylene ethers sulfonate (AES, 99%), Sodium dodecyl benzene sulfonate (SDBS, 99%), lauryl sodium sulfate (LSS, 99%), Dodecyl trimethyl ammonium bromide (DATB, 99%), ethyl acetate (99%), n-hexane (99%), and methylbenzene (99%) were all purchased from CHRON Chemicals (Chengdu, China). All chemicals received were not treated further. Deionized water was self-produced in the laboratory, and the electrical resistivity was greater than 18.2 MΩ. The coal rock samples are from the #8 coal seam of Nalin region in Shanxi formation in the permain system in Ordos basin, China. 

## 3. Methods

### 3.1. Synthesis of Silicon Quantum Dots (SQDs)

A total of 5.5 g of sodium citrate is dissolved in 80 g of deionized water. Then, this solution is deoxidized via filling N_2_ for 30 min, whereafter the weighed APTES is added in the above solution and stirred to prepare the precursor solution of SQD. Afterwards, the precursor solution is placed into a high-temperature and high-pressure reactor. Meanwhile, the temperature is set as 160 °C and the reaction time is 6 h. Finally, a dialysis bag of 1~3 kDa is employed to separate and purify the SQD. Then, the product is dried at 50 °C in the vacuum oven for 36 h. 

### 3.2. Synthesis of the Polycyclic Aromatic Hydrocarbon Monomer (AHM)

A 150 mL three-neck flask is placed in an ice-bath, which is filled with 50 mL of THF, and then, 0.25 mol of 9-phenanthrylamine and 0.2 mol of TEA are added. Subsequently, this solution is deoxidized via filling N_2_ for 25 min. A total of 10 mL of THF is used to dissolve 0.2 mol of acryloyl chloride, which is slowly added in the above three-neck flask. Afterwards, the reaction is carried out first in the ice-bath for 20 min and then at room temperature for 20 min. Finally, the reaction is finished and then the product is extracted by ethyl acetate, purified by n-hexane, and recrystallized by methylbenzene and n-hexane (*v*/*v* = 1:4). The reaction yield is 94%. Subsequently, the product is dried at 45 °C in a vacuum oven for 72 h.

### 3.3. Preparation of the Functional Polymer Modified Silicon Quantum Dots (PQS)

A total of 6 g of SQD is dispersed homogeneously in 90 mL of N,N-dimethyl acetamide and deoxidized via filling N_2_ for 40 min. Then, 0.6 g of AHM, 0.3 g of sulfobetaine methacrylate, 0.3 g of vinyl acetate, and 0.3 g of acrylic amide are added and stirred. A total of 0.3 g of potassium persulfate is dissolved in 10 mL of deionized water, which is slowly added in the above-mixed solutions. Then, the reaction is carried out at 70 °C for 8 h. Finally, the product is separated by high-speed centrifuge and washed by the deionized water.

### 3.4. Characterization

The infrared spectroscopy of the PQS in 4000–400 cm^−1^ is obtained by Fourier transform infrared spectrometer (FT-IR, Nicolet 5700, Waltham, MA, USA) at room temperature. The morphology of PQSs is acquired by transmission electron microscope (TEM, JEOL JEM-2100, Tokyo, Japan) to analyze their dispersing behavior and distribution of grain size. The surface zeta potential of PQSs is measured by the Zetasizer analyzer (ZETASIZER LAB, Malvern & PANalytical, Malvern, UK) at room temperature. The Darkroom UV analyzer and a spectrofluorophotometer (LS55, PerKinEImer, Waltham, MA, USA) are adopted to observe the fluorescence characteristic of the PQS fluid. The ultraviolet-visible spectrophotometer (UV-2600, SHIMADZU, Kyoto, Japan) is employed to evaluate the adsorption capacity of PQSs on the surface of coal rock at room temperature. The calibration curve of the PQS concentration is done firstly to calculate the adsorption capacity of PQS and the oscillation equilibrium method is used to realize the adsorption equilibrium of PQSs on the surface of coal powder. The formula for the static adsorption capacity is shown in the following Equation (1):

(1) $$\Gamma =\frac{V(c_{0}-c_{t})}{m}$$

Therein, Γ, the static adsorption capacity, mg/g; V, the volume of PQS nanofluid, mL; $c_{0}$, the initial concentration of PQS nanofluid, mg/L; $c_{t}$, the final concentration of PQS nanofluid after adsorption balance, mg/L; m, the weight of coal powder, g. All experiments are carried out three times and the average of these three values is taken as the final value. Then, all data are presented as the mean value ± standard deviation (SD).

### 3.5. The Contact Angle

The coal rock sample is cut by a linear cutting machine to coal slices with 40 mm diameter and 5 mm height. The polished coal slices are submerged completely in the prepared sample, which is aged for one week at room temperature. Then, the aged coal slice is taken out and dried at 55 °C for 12 h. The sessile drop method of interfacial tensiometer (DSA100, KRÜSS, Hamburg, Germany) is adopted to measure the contact angle of deionized water on the surface of original or aged coal samples at room temperature under atmospheric pressure. The contact angle is calculated by the software in the instrument. All experiments are carried out three times and the average of these three values is taken as the final value. Then, all data are presented as the mean value ± standard deviation (SD).

### 3.6. CBM Adsorption/Desorption Experiment

(1) Preparation of Coal Powder Samples

The raw coal rock is crushed by a pulverizer and then screened to prepare the coal powder with a particle size of 0.18–0.25 mm. Thereafter, the coal powder is dried by a vacuum oven at 55 °C for 12 h to remove free moisture and then vacuum packed for subsequent experiments.

(2) The Balanced Water Measurement

Before CBM adsorption/desorption experiment, the water should be balanced and the balanced water content should be measured. Meanwhile, the process is in accord with the standard of the American Society for Testing Materials (ASTM). The weighed coal powder is completely immersed in the deionized water for 2 h at 30 °C. Subsequently, the soaked coal powder is filtrated, and then the water is balanced for it. The balanced water content is obtained from the following Equation (2):

(2) $$M_{e}=\frac{m_{1}}{m_{2}}\times M_{ad}+(1-\frac{m_{1}}{m_{2}})\times 100$$

Therein, $M_{e}$, the balanced water content, %; $m_{1}$, the weight of air dried coal powder before balanced, g; $m_{2}$, the weight of coal powder after balanced water, g; $M_{ad}$, the water content of the air dried coal powder, %.

(3) Tightness Test

Firstly, the water balanced coal powder is used to fill in the sample cylinder and then the cylinder is sealed and loaded in the incubator. Then, the system temperature is set as the coal steam temperature.

Secondly, the helium is injected and the system pressure is made higher than the largest test pressure of 1~2 MPa. Then, the valves are closed and the pressures of the reference cylinder and sample cylinder are monitored.

Finally, keeping this state for at the least 6 h, if the pressure has no variation, the tightness of the equipment is considered to be good. If not, the joints of system should be checked.

(4) The Free Space Volume

The free space volume is the sum of the pore volume of the coal sample in the cylinder, which is equal to the difference in volume between the sample cylinder and coal sample. The experimental procedure is shown as follows:

Firstly, the temperature of the reference cylinder and sample cylinder is kept at the tested temperature.

Secondly, helium is injected and the pressure of the reference cylinder is kept at 2~3 MPa. Then, its valve is closed.

Thirdly, the valve between the reference cylinder and the sample cylinder is opened. When the pressure has no variation, the data needs to be recorded.

Finally, the above-mentioned processes are repeated 3 times until the difference value between two test results is less than 0.1 cm^3^. Then, the free space volume can be calculated. The formula is shown in the following Equations (3) and (4):

(3) $$V_{c}=\frac{\left(p_{2}\times V_{2}\right)/\left(Z_{2}\times T_{2}\right)+\left(p_{3}\times V_{3}\right)/\left(Z_{3}\times T_{3}\right)-\left(p_{1}\times V_{1}\right)/\left(Z_{1}\times T_{1}\right)}{p_{3}/\left(Z_{3}\times T_{3}\right)-p_{1}/\left(Z_{1}\times T_{1}\right)}$$

(4) $$V_{fs}=V_{0}-V_{c}$$

Therein, $V_{c}$, the volume of coal sample, cm^3^; $p_{2}$, the initial pressure of reference cylinder, MPa; $V_{2}$, the volume of reference cylinder, cm^3^; $Z_{2}$, the initial gas compression factor of the reference cylinder. $T_{2}$, the initial temperature of reference cylinder, K. $p_{3}$, the initial pressure of sample cylinder, MPa. $V_{3}$, the volume of sample cylinder, cm^3^; $Z_{3}$, the initial gas compression factor of the sample cylinder; $T_{3}$, the initial temperature of sample cylinder, K. $p_{1}$, the balanced pressure, MPa; $V_{1}$, the total volume of system, cm^3^; $Z_{1}$, the balanced gas compression factor; $T_{1}$, the balanced temperature, K. $V_{fs}$, the free space volume, cm^3^; $V_{0}$, the total volume of sample cylinder, cm^3^.

(5) The Isothermal Adsorption Experiment

An isothermal adsorption/desorption experimental instrument (designed and developed by Langfang Branch of Research Institute of Petroleum Exploration and Development, CNPC) is employed to carry out the isothermal adsorption experiment of CBM on the surface of coal steam with the influence of PQS and other components at a constant temperature of 25 °C. The experimental procedure is as follows: 

Firstly, the control valve of the reference cylinder is turned on and methane is adopted to fill the reference cylinder until the system pressure reaches the target pressure. 

Secondly, the valve jointed the coal sample cylinder is opened to start gas adsorption. The experimental data from the sample cylinder and the reference cylinder are collected. The ad-/desorption equilibrium time is set as 12 h and the maximum equilibrium pressure is set as 27.0 MPa. 

Finally, the tests are carried out from low pressure to high pressure and the number of pressure points is set based on the highest pressure. 

According to the above-mentioned method, the isothermal adsorption experiments of CBM on the surface of coal steam with the influence of PQS and other components are implemented. The formulas for calculating the adsorption capacity of CBM are shown as follows:

(5) pV=nZRT

(6) $$n_{i}=n_{1}-n_{2}$$

(7) $$V_{i}=n_{i}\times 22.4\times 1000$$

(8) $$V_{adsorption}=\frac{V_{i}}{G_{c}}$$

Therein, p, the gas pressure, MPa; V, the gas volume, cm^3^; n, the amount of substance for gas, mol; Z, the gas compressibility factor; R, molar gas constant, J·mol^−1^·K^−1^; T, the equilibrium temperature, K. And $n_{i}$, the amount of substance for the adsorption gas, mol; $n_{1}$, the amount of substance for the gas in sample cylinder before balancing, mol; $n_{2}$, the amount of substance for the gas in sample cylinder after balancing, mol. $V_{adsorption}$, the adsorption capacity of CBM, cm^3^/g; $V_{i}$, the total volume of the adsorption CBM, cm^3^; $G_{c}$, the weight of coal powder, g.

The Langmuir model, a monolayer adsorption equation, is usually employed to describe isothermal adsorption of CBM on the coal rock surface, which is based on the principle of dynamic equilibrium of adsorption and desorption. Therefore, the Langmuir pressure and Langmuir volume are obtained by the fitting curve of the isothermal adsorption. The calculated formula for them is derived from the Langmuir model and shown as follows:

(9) $$\frac{p}{V}=\frac{p}{V_{L}}+\frac{p_{L}}{V_{L}}$$

When $\frac{1}{V_{L}}$ is equal to A and $\frac{p_{L}}{V_{L}}$ is equal to B, the above equation can be changed as follows:

(10) $$\frac{p}{V}=Ap+B$$

Therefore,

(11) $$V_{L}=\frac{1}{A},P_{L}=BV_{L}$$

Therein, $V_{L}$, the max adsorption capacity, cm^3^/g; $p_{L}$, the Langmuir pressure, MPa, which is the pressure when the adsorption capacity of methane on the surface of coal rock is half of Langmuir volume. The Langmuir pressure is a key factor for characterizing the shape of the isothermal adsorption curve. The degree of curve bending is larger when the Langmuir pressure is lower. Instead, the smaller degree of curve bending is obtained when the Langmuir pressure is higher [29]. 

## 4. Results

### 4.1. Characteristic

#### 4.1.1. The Micro Properties Characteristic of PQS

**Figure 2:**
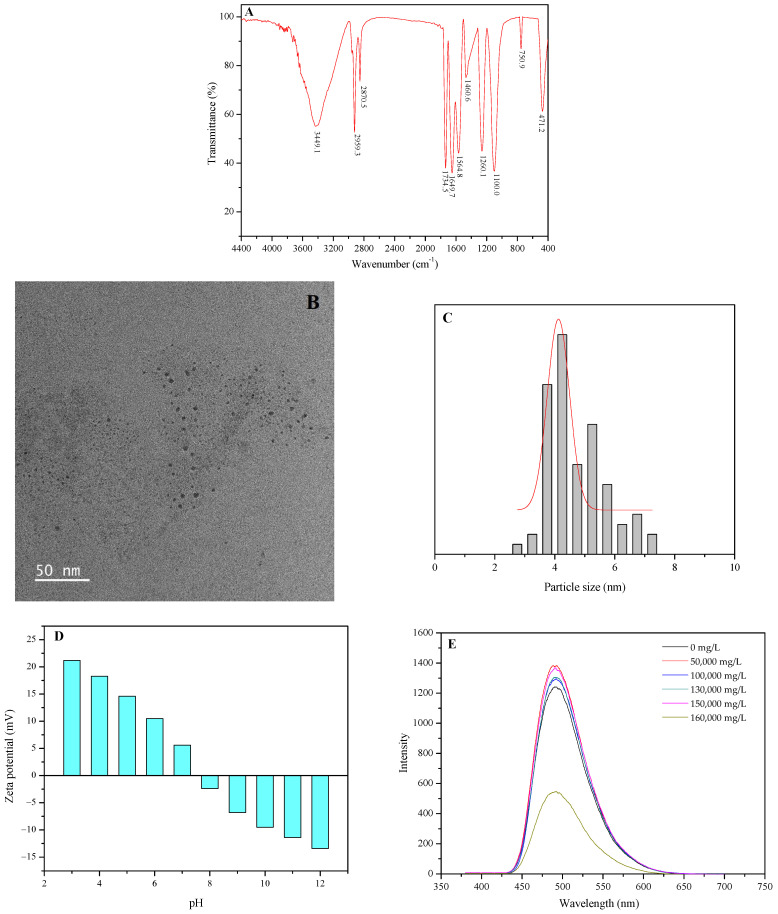
The micro-characteristic results of PQS. (**A**) The FT-IR spectrum of PQDs; (**B**) The micromorphology of PQDs obtained from a HRTEM; (**C**) The particle size distribution of PQDs analyzed by the software Image J 1.8.0; (**D**) The zeta potential of PQSs versus pH at room temperature; (**E**) The fluorescence characteristic of PQD nanofluid under room temperature.

The poly-aromatics-grafted silicon quantum dot-based desorption agent depends on many functional groups including aromatics, sulfonylbetain, oxygen-containing groups, and amino groups on its molecular structure to build a firm binding energy and strong adsorption capacity on the coal surface. The micro-characteristic results of PQS are shown as Figure 2. 

In Figure 2A, the FT-IR spectrum of PQDs has shown that the peak at 1734.5 cm^−1^ corresponds to the stretching vibration of C=O in vinyl acetate. The peak at 1649.7 cm^−1^ is the stretching vibration of C=O in acrylic amide. The points at 2959.3 cm^−1^ and 2870.5 cm^−1^ are the stretching vibration of -CH_3_ and -CH_2_, respectively. The peak at 1100.0 cm^−1^ is the symmetrical stretching vibration of Si-O-Si, which has proved the generation of the silicon quantum dot. The broad peak at 3449.1 cm^−1^ is the stretching vibration of –OH and N–H. The peak at 1460.6 cm^−1^ is the bending vibration of –CH_2_–. The peak at 1260.1 cm^−1^ corresponds to the symmetrical stretching vibration of S=O in –SO_3_−. The peaks at 1564.8 cm^−1^ and 750.9 cm^−1^ are the stretching vibration of phenanthrene ring. The peak at 471.2 cm^−1^ is the bending vibration of Si-O in the silicon quantum dot. Therefore, it can be considered that the silicon quantum dot has been successfully synthesized and the molecular structure of the polymer on its surface includes aromatics, sulfo-betaine, oxygen-containing groups, and cationic groups, which can bond with the coal surface via hydrogen bond, π–π stacking interaction, π–π conjugation, van der Waals, electrostatic attraction, and quantum size effect.

It can be found from Figure 2B that the silicon quantum dot still has a high crystallinity. Moreover, the micromorphology of PQDs mainly is globular or near-spheroidal and a small quantity of them are an ellipsoid or irregular shape. Meanwhile, an excellent dispersion has also been exhibited for PQDs. Then, Figure 2C shows that the particle sizes of PQDs are all lower than 10 nm and the median size is about 4.9 nm, which is completely met with the characteristic of quantum dot. Hence, under the injecting pressure, PQDs can be injected and migrate into the deeper place of the reservoir and the narrow pores due to the super-low size. Simultaneously, the forceful adsorption is formed on the surface of coal rock due to the quantum size effect. 

In Figure 2D, while the pH is increasing, the zeta potential of PQS has an obvious variation mainly due to the existence of amino. When the pH is equal to 3, in the protonation of amino, the zeta potential of PQS is equal to +21.2 mV, showing the strong electropositivity. When the pH is equal to 11, in the deprotonation of amino, the zeta potential of PQS has changed to weak electronegativity with −13.4 mV. Meanwhile, when the pH is neutral, in the protonation of amino, there is a weak electropositivity with +5.6 mV. Obviously, a strong adsorption of PQS on the surface of coal rock has been formed via electrostatic attraction in the acid and neutral condition, which is completely met with the coal reservoir environment in fracturing or acid fracturing. 

In Figure 2E, the PQS nanofluid has a significant emission peak with 470 nm at the excitation of 380 nm in the fluorescence emission spectrum and the intensity has reached 1382 A.U. Although the fluorescence characteristic of PQD nanofluid has no key influence on its application in the desorption agent of CBM, these results can be powerful evidence for the successful synthesis of silicon quantum dots. In addition, the fluorescence intensity of PQS nanofluid is stable with the increase in salinity, and when the salinity is 160,000 mg/L, there is an obvious decrease. It can be shown that the salinity resistance of PQS nanofluid is 150,000 mg/L.

#### 4.1.2. The Adsorption Capacity of PQSs on the Coal Surface

**Figure 3:**
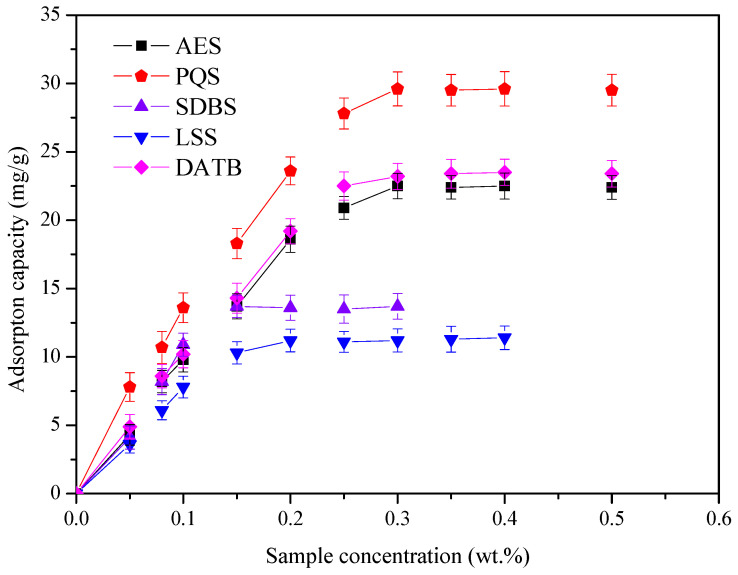
The adsorption capacity of PQS, SDBS, LSS, AES, and DATB on the coal surface.

The CBM is desorbed from the surface of coal rock by the desorption agent via forcefully competitive adsorption. Hence, the adsorption capacity of PQSs should be studied and the surfactants, including SDBS, LSS, AES, and DATB, are the comparative groups at room temperature. The results are shown in Figure 3.

It can be found that the adsorption capacity of all active chemicals first increase and are then stable with the increase in the used concentration. The stable stage is also called equilibrium adsorption concentration. Under this condition, the adsorption and desorption behavior of the active chemicals is in a state of dynamic equilibrium. Obviously, the PQS has the largest adsorption capacity with 29.6 mg/g, followed by the DATB with 23.2 mg/g, followed by the AES with 22.5 mg/g, followed by the SDBS with 13.7 mg/g, and finally followed by the LSS with 11.2 mg/g. The equilibrium adsorption concentrations of PQS, SDBS, LSS, AES, and DATB are 0.3%, 0.15%, 0.2%, 0.3%, and 0.3%, respectively. Therefore, the PQS has the best adsorption effect.

### 4.2. The Isothermal Adsorption/Desorption of CBM

#### 4.2.1. Isothermal Adsorption/Desorption Loop Curve

**Figure 4:**
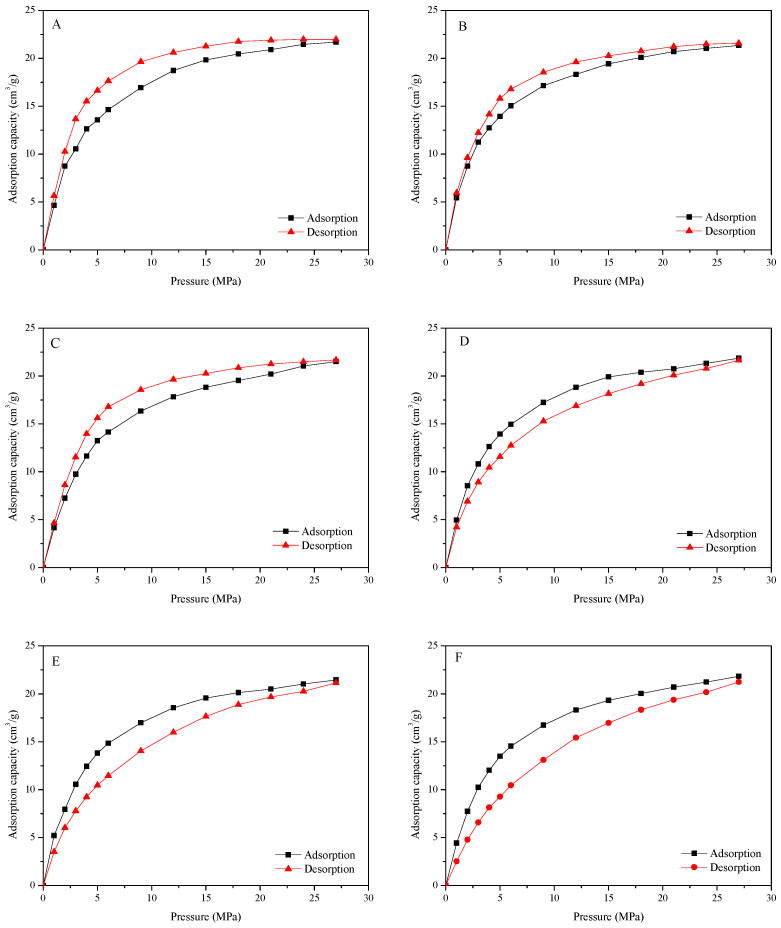
The isothermal adsorption/desorption loop curve of 0.3% DATB (**A**), and 0.3% LSS (**B**), 0.3% SDBS (**C**), 0.3% PQS (**D**), 0.3% AES (**E**), 0.3% PQS + 0.1% AES (**F**) treated coal samples.

The isothermal adsorption/desorption behavior of CBM on the coal surface with the influence of PQS and other active chemicals has been studied under 25 °C. The isothermal adsorption/desorption loop curves are shown in Figure 4.

Results show that the adsorption capacity of CBM increases with the increase in pressure and has no saturated adsorption in this pressure range. At the same time, there is an obvious hysteresis in the isothermal adsorption/desorption loop curve of SDBS, LSS, and DATB, the reason for which is the lag effect caused by the changes in the pore structure of coal as well as the generation and destruction of the Van der Waals forces during the adsorption/desorption of CBM. Instead, the adsorption capacity in the desorption process is lower than that in the adsorption process for the loop curves of PQS, AES and PQS + AES, which indicates that the adsorption capacity of CBM is weakened and part of the adsorbed gas has changed into free gas. Meanwhile, the amplitude of decrease for AES is greater than PQS but clearly less than AES + PQS. It can be considered that the influence of AES on the desorption of CBM is stronger than that of PQS and the synergy effect of PQS + AES can bring a significant promoting effect to PQS or AES fluid. 

#### 4.2.2. Langmuir Pressure and Langmuir Volume

**Figure 5:**
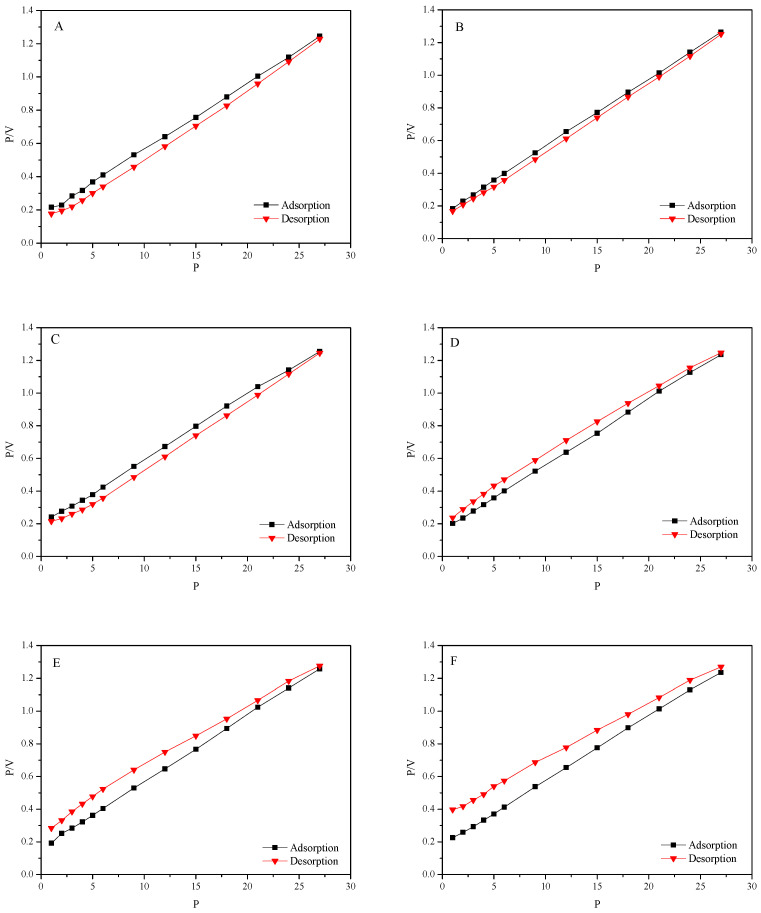
The isothermal adsorption/desorption line fitting curves for 0.3% DATB (**A**), and 0.3% LSS (**B**), 0.3% SDBS (**C**), 0.3% PQS (**D**), 0.3% AES (**E**), 0.3% PQS + 0.1% AES (**F**) treated coal samples.

According to the Langmuir model, the isothermal adsorption/desorption curve is fit by the linear least square method to obtain the linear equation of P/V~P. Then, the slope and intercept are obtained to calculate the Langmuir pressure and Langmuir volume. During the isothermal desorption, the promoting or inhibiting action of the active chemicals has been characterized by these two parameters. When the Langmuir pressure is increased, the isothermal desorption of CBM is promoted. Conversely, the isothermal desorption of CBM is inhibited. The fitted P/V~P linear equation curves for PQS + AES, PQS, SDBS, LSS, AES, and DATB treated coal samples are shown in Figure 5. Moreover, the calculated results of Langmuir pressure and Langmuir volume are presented in Table 1. 

The results show that the Langmuir volume and the Langmuir pressure are all decreased for SDBS, LSS, DATB treated coal samples, which has an inhibiting action on the desorption of CBM. The volume of desorption gas per unit pressure difference is reduced during isothermal desorption. However, the Langmuir volume and the Langmuir pressure are all increased for PQS, AES, and PQS + AES treated coal samples, which has an obviously promoting action to the desorption of CBM. The volume of desorption gas per unit pressure difference is increased. In Table 1, the $∆P_{L}$ with −66.33% corresponding to DATB is greater than that corresponding to SDBS and LSS. Thus, the DATB treated coal sample has a more severe inhibiting action than SDBS and LSS. Nevertheless, $∆P_{L}$ with 46.93% corresponding to AES is larger than that with 32.7% corresponding to PQS but obviously lower than that with 57.28% corresponding to PQS + AES. It can be considered that PQS + AES has an excellent promoting action for desorption of CBM in the synergy effect. 

#### 4.2.3. The Desorption Capacity of CBM

**Figure 6:**
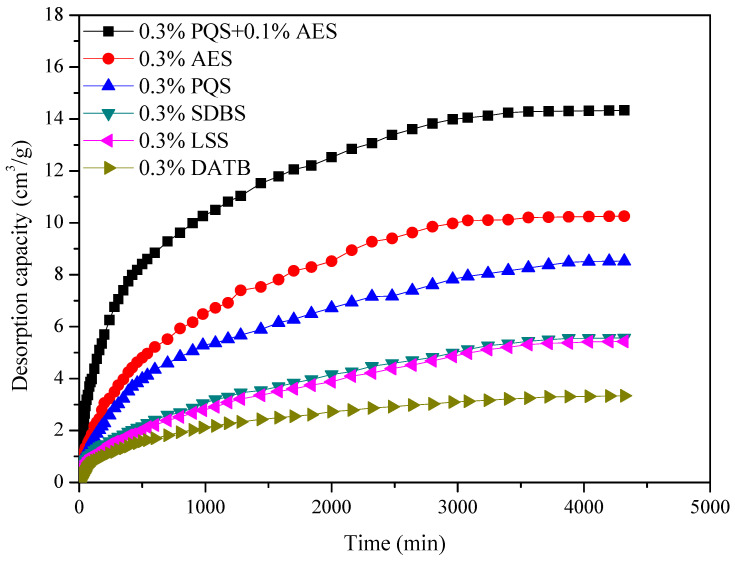
The desorption rate of CBM with the influence of 0.3% PQS + 0.1% AES, 0.3% AES, 0.3% PQS, 0.3% SDBS, 0.3% LSS, and 0.3% DATB.

According to the reservoir characterizing parameters of the #8 coal seam in the Nalin region, the desorption experiment of CBM with the influence of PQS has been done. The active chemical fluids are injected into the sample cylinder until the pressure reaches 45 MPa and maintained for 2 h, which simulates the fracturing process. Then, the pressure in the sample cylinder is reduced slowly to the reservoir pressure with 27 MPa, whereafter the desorption of CBM is started and this process is implemented for 72 h. The experimental temperature, set as the reservoir temperature at 90 °C, is obtained by an oil-bath. The desorption capacity of CBM with the influence of 0.3% DATB and 0.3% PQS, 0.3% SDBS, 0.3% LSS, 0.3% AES, 0.3% PQS + 0.1% AES versus time is shown in Figure 6.

After 72 h of desorption, it can be found that the desorption capacity of CBM is increased with the passage of time. Obviously, the PQS + AES treated coal sample has the highest desorption capacity of CBM with 14.26 cm^3^/g, followed by PQS with 10.25 cm^3^/g, followed by AES with 8.52 cm^3^/g, followed by SDBS with 5.56 cm^3^/g, followed by LSS with 5.42 cm^3^/g, followed by DATB with 3.29 cm^3^/g. In these experimental conditions, the saturated adsorption capacity of CBM is 21.85 cm^3^/g. Therefore, the calculated desorption rates for PQS + AES, PQS, AES, SDBS, LSS, DATB, are 65.3%, 46.9%, 39.0%, 25.5%, 24.8%, and 15.1%, respectively. Significantly, the desorption agent using modified silicon quantum dot is higher than the conventional surfactant fluid for enhancing the recovery of CBM. Furthermore, the results show that there is a good synergy effect between PQS and AES for desorption of CBM.

#### 4.2.4. Wettability Alteration on the Coal Surface

**Figure 7:**
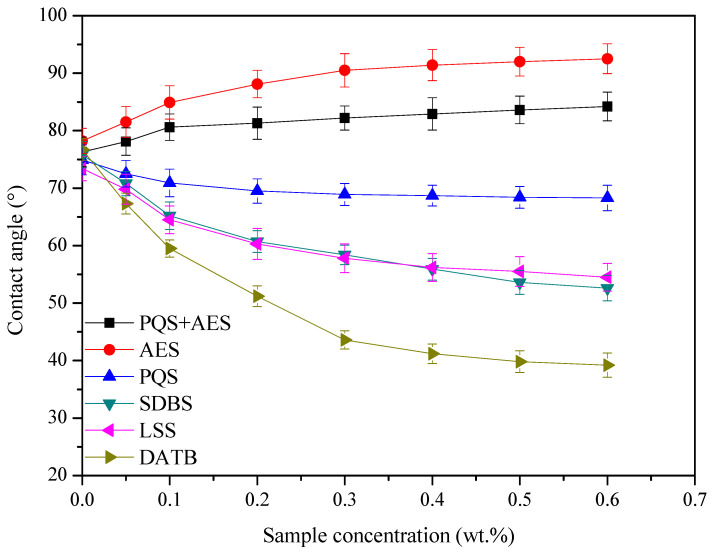
The water contacts angle of coal slices with and without the influence of PQS + AES, AES, PQS, SDBS, LSS, and DATB.

Wettability, as a vital parameter, indicates the competitive adsorption result of the active chemicals on the surface of coal rock and mobility of CBM. The hydrophobicity is stronger, the capillary resistance is lower and the flow capacity of CBM is better. The water contact angles of coal slices with and without the influence of PQS + AES, AES, PQS, SDBS, LSS, and DATB versus the corresponding sample concentration are shown in Figure 7.

It can be found that with the increase in the sample concentration, the water contact angle on the surface of coal slice has a clear decrease for SDBS, LSS, DATB, and PQS. Simultaneously, it has an increase of contact angle in the effect of AES. The initial contact angle for the coal slice shows closely neutral wettability in the range of 70° to 80°. After treatment, the final contact angle of the coal slice is changed to 92.5°, 68.3°, 52.6°, 54.5°, and 39.2° for AES, PQS, SDBS, LSS, and DATB, respectively. Therefore, stronger hydrophilicity is generated for the wettability of the coal slice as an effect of PQS, SDBS, LSS, and DATB. Meanwhile, the hydrophobicity on the surface of the coal rock is obviously enhanced by AES, so the mobility of CBM is improved. However, when AES is added to the 0.3% PQS dispersed fluid, the hydrophobicity alteration for the coal-rock surface shows their synergy effect, and the final contact angle is 84.2°. That is to say, the hydrophobicity alteration of PQS for the coal rock surface is enhanced by AES, which is a benefit of the flow-out of CBM.

## 5. Discussion

According to the above-studied results, PQS nanofluid has an excellent desorption effect for CBM in #8 coal seam in Nalin region, especially synergizing with anionic–nonionic surfactant AES. The competitive adsorption and wettability alteration effect of desorption agent to the coal-rock surface are closely related to the intermolecular interaction between them. 

The vitrinite reflectance is greater than 1.8% and the coal rank is medium-high rank meager coal for the #8 coal seam in Shanxi formation in Nalin region. The mineral composition includes more than 80% of organics containing a large amount of aromatics and bits of inorganics, mainly clay. Therefore, the intermolecular force between methane and coal surface is mainly the Van der Waals force and the dispersion force plays a key role for the bond of C-H and aromatic molecules. Furthermore, the electrostatic effect and indirect hydrogen bonding have a weak contribution to the bond between C-H with charged groups and –OH, respectively [30].

DATB, a cationic surfactant, has a NH_4_^+^ in its hydrophilic group. Hence, a strong electrostatic attraction is built to cause high adsorption capacity when DATB is met with the negative charge of oxygen-containing groups (–COO^−^, Ar-OH, etc.) on the surface of coal rock. In this study, the high concentration of DATB is used, so the double-layer adsorption or micelle adsorption has been formed for DATB on the coal surface. The hydrophilic groups face outward to cause strong hydrophilicity on the coal surface, which generates a large number of adsorption sites again to inhibit desorption of CBM [31].

LSS, an anionic surfactant, has a SO_4_^−^ in its hydrophilic group. Thus, electrostatic repulsion is generated between LSS and the negatively charged groups on the coal surface to prevent adsorption of LSS, which results in a sparse arrangement of hydrophobic groups. However, a strong hydrogen-bonding effect between LSS and the oxygen-containing groups on the coal surface and Van der Waals force are generated to enhance competitive adsorption of LSS. The comprehensive adsorption capacity for LSS is weaker than DTAB, but the hydrophilicity of coal surface is improved as an effect of LSS [32].

SDBS, an anionic surfactant, has an Ar–SO_3_^−^ in its hydrophilic group. Thus, compared with LSS, apart from hydrogen bonding, Van der Waals force, and electrostatic repulsion, the π–π stacking interaction and π–π conjugate effect are generated between the aromatic of SDBS and aromatics on the coal surface. It can be found that SDBS has a slightly stronger adsorption capacity with the coal surface than LSS but still weaker than DATB. Therefore, an improved hydrophilicity for coal surface is obtained to prevent desorption of CBM [33].

AES, an anionic–nonionic surfactant, has many O–H bonds in its nonionic hydrophilic group. Hence, apart from Van der Waals force and electrostatic repulsion, the hydrogen bonding effect plays a key role in the adsorption of AES on the surface of coal rock. The competitive adsorption capacity for AES is obviously higher than SDBS and LSS. Meanwhile, the sulfonic acid group is anchored on the coal surface but the polyoxy-ethylene chain and long hydrocarbon chain face outward to cause the enhanced hydrophobicity of the coal surface and a strong promoting effect for desorption of CBM [34].

As for PQS, a self-developed polymer-grafted silicon quantum dot, the results from its FT-IR and Zeta potential have proved that aromatic, oxygen-containing groups, cationic groups exist on the quantum dot surface. Moreover, the median particle size of PQDs is about 4.9 nm, which has an approximately 10~20 times higher specific surface energy than conventional SiO_2_ nanoparticles to promote the adsorption of PQD [35]. Further precise calculations for the specific surface energy need to be measured by the BET method. Therefore, PQSs have a very strong competitive adsorption capacity on the coal surface in the synergistic effect of π–π stacking interaction, π–π conjugation, electrostatic attraction, Van der Waals force, hydrogen bonding effect and excess quantum size effect, which causes a slightly improved hydrophilicity of the coal surface. However, PQS has a promoting effect to the desorption of CBM.

PQS + AES, a mixture with PQS and anionic–nonionic surfactant AES, in addition to the competitive adsorption capacity of them on the coal surface via intermolecular force, show a synergistic effect to generate an excess osmotic pressure to promote the separation of gas from the coal surface. The osmotic pressure is generated by the concentration difference of nanofluid between the inner and outer sides of the wedge region [24,25]. Then, a slightly enhanced hydrophobicity for the coal surface and a significant desorption effect for CBM are obtained by this synergy effect. This study provides guidance for the molecular design of the desorption agent in deep coal seam and the application of silicon quantum dots.

## 6. Conclusions

To enhance the recovery of CBM in #8 coal seam in Shanxi formation in Nalin region, this paper has developed a poly-aromatics grafted silicon quantum dot, which synergizes with an anionic–nonionic surfactant to promote desorption of CBM. Then, the adsorption/desorption behavior of CBM on the coal surface with the influence of these active chemicals has been studied. The obtained conclusions are as follows:

(1) The designed poly-aromatics-grafted silicon quantum dot has the characteristic of quantum dot nano-material and the functional groups on its surface have a strong adsorption capacity with the coal surface via the intermolecular forces including hydrogen bonding, quantum size effect, electrostatic attraction, Van der Waals force, π–π stacking interaction, and π–π conjugate effect. The salinity resistance of PQS nanofluid is 150,000 mg/L, which is very well-suited for application in #8 coal seam.
(2) Compared with the conventional ionic surfactants, PQS nanofluid has a promoting effect to the escape of CBM. Especially, when PQS nanofluid is synergized with anionic–nonionic surfactant AES, the Langmuir pressure is significantly increased for the enhancement of desorption of CBM and the desorption rate of CBM reaches 65.3%.
(3) Compared with the conventional desorption agent for the natural gas in sand rock, apart from Van der Waals force, hydrogen bonding, and electrostatic effect, the π–π stacking interaction and π–π conjugation play a key role for the competitive adsorption of desorption agent on the coal rock surface. Meanwhile, the use of the size effect and the synergy effect between nanofluid and surfactant fluid to combine with the traditional intermolecular force can obviously enhance the promoting effect to the desorption of CBM. This study provides guidance for the molecular design of the desorption agent in deep coal seam and the application of silicon quantum dots.

## Figures and Tables

**Table 1:** The calculated results of Langmuir volume and Langmuir pressure for 0.3% DATB, and 0.3% LSS, 0.3% SDBS, 0.3% PQS, 0.3% AES, 0.3% PQS + 0.1% AES treated coal samples.

Active Chemicals	Process	V_L_ (cm^3^/g)	P_L_ (MPa)	ΔV_L_ (%)	ΔP_L_ (%)
0.3% DATB	Adsorption	25.06	4.12	−2.76	−66.33
	Desorption	24.39	2.48		
0.3% LSS	Adsorption	24.15	3.57	−0.72	−28.73
	Desorption	23.98	2.78		
0.3% SDBS	Adsorption	25.13	4.81	−2.01	−45.64
	Desorption	24.63	3.31		
0.3% PQS	Adsorption	24.88	3.92	3.23	32.7
	Desorption	25.71	5.83		
0.3% AES	Adsorption	24.51	3.91	7.6	46.93
	Desorption	26.53	7.37		
0.3% PQS + 0.1% AES	Adsorption	25.25	4.51	13.38	57.28
	Desorption	29.15	10.56		

## Data Availability

All data generated or analyzed during this study are included in this published article.
